# Reliability and Validation of Three Scoring Systems in the Assessment of Diabetic Foot Ulcers

**DOI:** 10.7759/cureus.106171

**Published:** 2026-03-30

**Authors:** Mruthunjaya S Bevinamarad, Ramakanth Baloorkar, Shivanagouda S Patil, Shailesh Kannur, Kanishk Kanishk, Nagaraj Biradar, Bhuvaneshwari Gachinmath

**Affiliations:** 1 General Surgery, Shri BM Patil Medical College, Hospital and Research Centre, BLDE (Deemed to be University), Vijayapura, IND

**Keywords:** amputation, diabetic foot ulcers, diabetic ulcer severity score, sinbad, university of texas

## Abstract

Introduction

Diabetic foot ulcers represent a major cause of morbidity and are frequently associated with infection, delayed healing, and limb loss. Accurate risk stratification using standardized scoring systems is essential for predicting outcomes and guiding management decisions. The present study aimed to evaluate and compare three ulcer scoring systems (Diabetic Ulcer Severity Score (DUSS), Site, Ischemia, Neuropathy, Bacterial Infection, Area, and Depth (SINBAD) scoring, and University of Texas classification) in predicting clinical outcomes, specifically secondary healing, requirement of split-thickness skin grafting (STSG), and lower limb amputation, among patients with diabetic foot ulcers.

Materials and methods

This prospective observational study included 107 patients aged ≥35 years with diabetic foot ulcers treated at a tertiary care centre over two years. Ulcers were assessed at baseline using the Diabetic Ulcer Severity Score, the University of Texas (UT) classification, and the SINBAD scoring system. Patients were followed until a definitive outcome. Clinical outcomes were categorized as secondary healing, requirement of split-thickness skin grafting, or lower limb amputation. Non-parametric statistical analysis was performed using the Kruskal-Wallis test, and a p-value <0.05 was considered statistically significant.

Results

The mean age of participants was 56.84 ± 12.32 years, with a male predominance (84, 78.3%). Most ulcers were located in the midfoot (45, 42.1%) and forefoot (44, 41.1%). STSG was required in 84 (78.5%) patients, amputation was performed in 18 (16.8%), and secondary healing occurred in five (4.7%). DUSS and UT classification did not show statistically significant differences across outcome groups. In contrast, SINBAD scores were significantly higher in the amputation group (median 5, IQR 4-6) compared to the STSG group (median 4, IQR 3-5) and secondary healing group (median 2, IQR 1-2) (H=8.02, p=0.018), demonstrating better discriminatory ability.

Conclusion

Among the evaluated scoring systems, SINBAD showed superior predictive performance for adverse clinical outcomes and may serve as a practical tool for risk stratification in diabetic foot ulcer management.

## Introduction

A diabetic foot ulcer (DFU) is defined as an ulceration of the foot associated with neuropathy, varying degrees of ischemia, and infection [1]. It is estimated that around 19% to 34% of patients with diabetes may develop a foot ulcer during their lifetime [2]. Diabetic foot ulcer represents one of the most serious and debilitating complications of diabetes mellitus [2]. The five-year risk of mortality for a patient with a diabetic foot ulcer is 2.5 times higher than that for a patient without a foot ulcer [2]. With such a high level of morbidity resulting from debilitating osteomyelitis and amputation in patients with DFU, it is of utmost importance to properly address and treat the underlying causes of the condition [3].

The sites of ulcers in diabetes are typically located in areas of the foot exposed to constant pressure or repetitive trauma [4]. Diabetic foot ulcers arise from a complex interplay of factors such as prolonged duration of diabetes, poor glycaemic control, inadequate foot care, structural foot deformities, dry skin, and repeated shear stress on the diabetic foot, leading to callus formation and subsequent ulceration [5]. These ulcers not only compromise quality of life but also significantly increase the risk of infection, hospitalization, and lower limb amputation [6].

Early identification of high-risk patients is therefore critical, and simple screening tools used by primary care physicians and paramedical staff can play a pivotal role in reducing morbidity and mortality [7]. Several scoring systems, including the Diabetic Ulcer Severity Score (DUSS), the Site, Ischemia, Neuropathy, Bacterial Infection, Area, and Depth (SINBAD) classification, and the University of Texas wound classification system (UT classification), have been developed to assess ulcer severity and predict outcomes [8-10]. Comparing these scoring systems can provide valuable insights into their relative effectiveness in forecasting wound healing in patients with DFU [11]. An accurate and reliable predictive score is essential for timely intervention, guiding appropriate management, and ultimately preventing progression to severe complications, including lower limb amputation [12].

The present study aimed to evaluate and compare three ulcer scoring systems (DUSS, SINBAD, and UT classification) in predicting clinical outcomes, specifically secondary healing, requirement of split-thickness skin grafting (STSG), and lower limb amputation, among patients with diabetic foot ulcers.

This research work was originally conducted as part of a postgraduate dissertation submitted to the Department of Surgery, BLDE (Deemed to be University), Vijayapura, Karnataka, India.

## Materials and methods

This prospective observational study was conducted in the Department of General Surgery at Shri BM Patil Medical College Hospital and Research Centre, Vijayapura, over a period of two years from January 2024 to December 2025. The institution is a tertiary care teaching hospital catering to both urban and rural populations. All patients presenting with diabetic foot ulcers (DFU) to the surgical outpatient department (OPD) and those admitted to the inpatient department (IPD) during the study period were screened for eligibility. Consecutive eligible patients were enrolled in order to minimize selection bias. Institutional Ethics Committee approval was obtained prior to commencement of the study (Approval No. BLDE(DU)/IEC-SBMPMC/094/2023-24). The study was conducted in accordance with ethical standards, and written informed consent was obtained from all participants before inclusion.

The sample size was calculated based on the proportion of amputation observed in patients with diabetic foot ulcers as reported by Satihal SN et al., in which 4.35% of patients underwent amputation during follow-up [12]. Considering a 95% confidence level (Z=1.96) and an absolute precision of 4%, the required sample size was determined using the single population proportion formula n = Z² × p × (1 − p) / d², where p represents the expected proportion (0.0435) and d represents the margin of error (0.04). Substituting these values yielded a calculated sample size of approximately 100 participants. Accounting for potential dropouts and incomplete follow-up, the final sample size was increased by 7%, resulting in 107 participants.

Patients aged ≥35 years with a confirmed diagnosis of diabetes mellitus presenting with diabetic foot ulcers were included in the study. Patients receiving long-term systemic steroid therapy and those who were immunocompromised, including individuals with HIV infection, malignancy undergoing chemotherapy, or other conditions affecting immune status, were excluded to avoid confounding factors that could influence wound healing outcomes.

A structured case record proforma was used to collect demographic and clinical data. Details recorded included age, sex, duration of diabetes, glycaemic control status, associated comorbidities, and relevant past medical history. A detailed local examination of the affected limb was performed in each patient. Ulcers were assessed for site, size, and number. Ulcer size was measured in centimetres using a sterile measuring scale by recording the maximum length and breadth. The presence of discharge, necrotic tissue, gangrene, and clinical signs of infection was documented.

Each ulcer was evaluated at baseline using three validated scoring systems: the Diabetic Ulcer Severity Score (DUSS), the University of Texas wound classification system (UT classification), and the SINBAD classification [8-10]. The DUSS includes four binary parameters: absence of pedal pulses, probing to bone, ulcer location (foot versus toe), and presence of multiple ulcers, each assigned a score of 0 or 1, yielding a total score ranging from 0 to 4 [8]. The University of Texas classification categorizes ulcers based on depth (Grades 0 to 3) and the presence or absence of infection and ischemia (Stages A to D), providing a combined grading system [10]. The SINBAD score evaluates six variables, namely site, ischemia, neuropathy, bacterial infection, area, and depth, each scored as 0 or 1, resulting in a total score ranging from 0 to 6 [9]. All scoring was performed at initial presentation prior to definitive intervention. All ulcer scoring was performed by a single trained surgeon to minimize inter-observer variability. Management decisions were made according to institutional treatment protocols and were not influenced by the study scoring.

Patients were followed up clinically until a definitive outcome was achieved. The primary outcome measure was wound healing status, while secondary outcomes included the requirement of STSG and lower limb amputation. Secondary healing was defined as complete wound closure without the need for surgical intervention such as grafting or amputation.

Data were entered into Microsoft Excel and analysed using Statistical Package for the Social Sciences (SPSS) version 26 (IBM Inc., Armonk, New York, USA). Continuous variables were expressed as mean ± standard deviation for normally distributed data and as median with interquartile range for non-normally distributed data. Due to non-normal distribution and unequal group sizes, the non-parametric Kruskal-Wallis test was used for comparisons among more than two groups. A p-value of less than 0.05 was considered statistically significant.

## Results

The study included 107 patients with diabetic foot ulcers, with a mean age of 56.84 ± 12.32 years (range 36-85 years). Males predominated, accounting for 84 (78.3%), while females comprised 23 (21.7%). The median ulcer size was 9.00 cm² (IQR 10.0). Most patients presented with a single ulcer 90 (84.1%), whereas multiple ulcers were observed in 17 (15.9%) cases (Table 1).

**Table 1:** Baseline demographic and ulcer characteristics (n=107) Data are presented as mean ± standard deviation (SD), range, median with interquartile range (IQR), or number (percentage), as appropriate. Descriptive statistics were used to summarize baseline demographic and ulcer characteristics.

Variable	Statistic	Value
Age (years)	Mean ± SD	56.84 ± 12.32
	Range	36–85
Gender	Male	84 (78.3%)
	Female	23 (21.7%)
Ulcer Size (cm²)	Median (IQR)	9.00 (10.0)
	Minimum-Maximum	1.00-96.00
Number of Ulcers	Single	90 (84.1%)
	Multiple	17 (15.9%)

Ulcers were most frequently located in the midfoot 45 (42.1%) and forefoot 44 (41.1%), together accounting for more than four-fifths of cases. Hindfoot involvement was less common eight (7.5%). Mixed-site patterns were relatively infrequent, with forefoot predominance over midfoot seen in seven (6.5%) and midfoot predominance over hindfoot in three (2.8%) patients (Table 2).

**Table 2:** Distribution of ulcer site (n=107) Data are presented as number (percentage). Descriptive statistics were used to summarize the anatomical distribution of ulcer sites among study participants. "forefoot > midfoot" indicates ulcers involving both the forefoot and midfoot with predominant involvement of the forefoot, while "midfoot > hindfoot" indicates ulcers involving both the midfoot and hindfoot with predominant involvement of the midfoot.

Ulcer site	n	%
Midfoot	45	42.1
Forefoot	44	41.1
Hindfoot	8	7.5
Forefoot > midfoot	7	6.5
Midfoot > hindfoot	3	2.8
Total	107	100.0

With respect to clinical outcomes, the majority of patients required split-thickness skin grafting (STSG), 84 (78.5%). Amputation was performed in 18 (16.8%) patients, whereas complete secondary healing without grafting occurred in five (4.7%) cases, indicating that most ulcers required surgical intervention for definitive management (Table 3).

**Table 3:** Clinical outcomes (n=107) Data are presented as number (percentage). Descriptive statistics were used to summarize clinical outcomes of diabetic foot ulcers in the study population.

Outcome	n	%
Secondary healing	5	4.7
Amputation	18	16.8
Split-thickness skin grafting (STSG)	84	78.5
Total	107	100.0

On non-parametric comparison using the Kruskal-Wallis test, DUSS scores did not differ significantly across outcome groups (H=0.11, df=2, p=0.94). Similarly, the University of Texas classification showed no statistically significant association with outcomes (H=4.36, df=2, p=0.11). In contrast, SINBAD scores demonstrated a statistically significant difference across outcome groups (H=8.02, p=0.018), with median scores of five (IQR 4-6) in the amputation group, four (IQR 3-5) in the STSG group, and two (IQR 1-2) in the secondary healing group, indicating better discriminatory ability for predicting adverse outcomes (Table 4).

**Table 4:** Association of scoring systems with clinical outcomes (Kruskal-Wallis Test) Data are presented as median with interquartile range (IQR). Comparisons among outcome groups were performed using the Kruskal-Wallis test. A p-value < 0.05 was considered statistically significant. DUSS - Diabetic Ulcer Severity Score; SINBAD - Site, Ischemia, Neuropathy, Bacterial infection, Area and Depth score; STSG - split-thickness skin grafting

Scoring system	Secondary healing (n=5), median (IQR)	Amputation (n=18), median (IQR)	STSG (n=84), median (IQR)	H statistic (χ²)	df	p-value
DUSS	1 (1-2)	3 (2-4)	3 (2-4)	0.11	2	0.94
University of Texas	2 (2-2)	3 (2-4)	3 (2-4)	4.36	2	0.11
SINBAD	2 (1-2)	5 (4-6)	4 (3-5)	8.02	2	0.018

## Discussion

The demographic profile observed in this cohort reflects the established epidemiology of diabetic foot disease. The mean age of 56.84 ± 12.32 years and male predominance (84, 78.3%) are consistent with previously published data indicating a higher incidence of diabetic foot ulcers among middle-aged to elderly men [13-15]. Leese et al. reported a mean age of 65.4 ± 4 years with 768 (72%) male participants, while George et al. documented a mean age of 58.9 ± 10.2 years with male predominance [16,17]. Similar gender distribution was described by Jayalal et al., who reported 95 (63%) male involvement [18]. The male predominance may reflect greater occupational exposure to repetitive trauma, delayed healthcare-seeking behavior, and higher prevalence of peripheral vascular disease in men.

Ulcer characteristics in the present study demonstrated wide variability in size (1.00-96.00 cm²), with a median area of 9.00 cm². Most patients presented with a single ulcer (90, 84.1%), and lesions were predominantly located in the midfoot (45, 42.1%) and forefoot (44, 41.1%). These anatomical distributions correspond to pressure-bearing and shear-stress zones, consistent with the established pathophysiology of neuropathic ulceration [19]. Similar anatomical predominance of forefoot and plantar lesions has been reported by Jayalal et al., reinforcing the relationship between mechanical stress and ulcer development [18].

With regard to prognostic performance, the present findings demonstrated that DUSS and UT classification did not show statistically significant differences across outcome groups, whereas SINBAD scores were significantly associated with clinical outcomes. The median SINBAD score increased progressively from secondary healing to STSG and amputation groups (p=0.018), suggesting superior discriminatory ability. Ince et al., who originally developed the SINBAD system, demonstrated that increasing SINBAD scores were strongly associated with impaired healing and adverse outcomes [9]. Leese et al. further validated SINBAD in a large cohort and reported a c-statistic of 0.72, comparable to or superior to more complex classification systems [16]. The superior performance of the SINBAD score in the present study may be attributed to its inclusion of ischemia and ulcer area, which are critical determinants of wound healing and amputation risk in tertiary care settings. These parameters directly reflect vascular compromise and tissue burden, which often guide surgical decision-making, thereby enhancing its discriminatory ability compared to DUSS and UT classification. The relative simplicity of SINBAD, incorporating ischemia, infection, neuropathy, and ulcer dimensions, may enhance its predictive robustness across diverse clinical settings.

In contrast, although DUSS has been shown to correlate with healing probabilities in earlier studies, including those by Beckert et al., its discriminatory performance in the present cohort was limited [8]. One possible explanation is the restricted score variability observed, as no patient had a DUSS score of 0, indicating that most ulcers presented with at least one adverse severity parameter. The absence of DUSS score 0 cases suggests referral bias and reduced score dispersion, which may have attenuated discriminatory performance. Similarly, the UT classification, while widely used and clinically intuitive, did not demonstrate statistically significant differences in this study. Previous literature has shown variable performance of UT staging depending on population characteristics and prevalence of ischemia and infection [20].

The higher SINBAD scores observed among patients undergoing amputation in this study are clinically meaningful. Amputation risk in diabetic foot disease is closely linked to the combined effects of ischemia, infection, ulcer depth, and surface area parameters directly incorporated within the SINBAD framework. Systematic reviews have emphasized that multidimensional scoring systems that integrate vascular and infectious parameters outperform depth-only classifications in predicting limb loss [18,21]. Therefore, the significant association observed supports the practical applicability of SINBAD in routine clinical risk assessment.

Strengths and limitations

This study has several important strengths. It prospectively evaluated three widely used diabetic foot ulcer scoring systems within the same clinical cohort, allowing direct comparison of their prognostic performance under uniform treatment conditions. The use of standardized scoring at baseline and clearly defined clinical outcomes enhances internal validity and reproducibility. Additionally, the inclusion of objective statistical analysis using non-parametric methods strengthens the robustness of outcome comparisons. However, certain limitations must be acknowledged. The study was conducted at a single tertiary care center, which may limit generalizability to primary care or community settings. The relatively small number of patients in the secondary healing group may have reduced statistical power for subgroup comparisons. Furthermore, referral bias toward more severe ulcers, reflected by the absence of DUSS score 0 cases, may have influenced the discriminatory performance of some scoring systems. Additionally, potential inter-observer variability in ulcer assessment may have influenced scoring accuracy, although efforts were made to standardize evaluation. Larger multicentric studies with longer follow-up are warranted to further validate these findings across diverse populations.

## Conclusions

Among the three evaluated scoring systems, the SINBAD classification demonstrated superior discriminatory ability in predicting clinical outcomes in patients with diabetic foot ulcers, particularly in identifying those at higher risk for amputation. While the Diabetic Ulcer Severity Score (DUSS) and the University of Texas classification remain useful descriptive tools, their predictive performance was limited in this cohort. The findings underscore the importance of structured risk stratification in routine clinical practice and suggest that the SINBAD scoring system may serve as a practical, reliable, and easily applicable tool for guiding management decisions and improving outcome prediction in patients with diabetic foot ulcers. However, these findings should be interpreted with caution, given the single-center design, and further large-scale multicentric studies are warranted to validate these results.
